# Population level determinants of acute mountain sickness among young men: a retrospective study

**DOI:** 10.1186/1471-2458-11-740

**Published:** 2011-09-28

**Authors:** Xiaoxiao Li, Fasheng Tao, Tao Pei, Haiyan You, Yan Liu, Yuqi Gao

**Affiliations:** 1 Department of Health Service, College of High Altitude Military Medicine, Third Military Medical University, 30 Gaotanyan Street, Shapingba District, Chongqing, China; 2 Key Laboratory of High Altitude Medicine, Ministry of Education, Third Military Medical University, 30 Gaotanyan Street, Shapingba District, Chongqing, China; 3 Urumqi General Hospital of Xinjiang Military District, Urumqi, Xinjiang, China

**Keywords:** acute mountain sickness, risk factor, high altitude, young men, logistic regression, retrospective study

## Abstract

**Background:**

Many visitors, including military troops, who enter highland regions from low altitude areas may suffer from acute mountain sickness (AMS), which negatively impacts workable man-hours and increases healthcare costs. The aim of this study was to evaluate the population level risk factors and build a multivariate model, which might be applicable to reduce the effects of AMS on Chinese young men traveling to this region.

**Methods:**

Chinese highland military medical records were used to obtain data of young men (n = 3727) who entered the Tibet plateau between the years of 2006-2009. The relationship between AMS and travel profile, demographic characteristics, and health behaviors were evaluated by logistic regression. Univariate logistic models estimated the crude odds ratio. The variables that showed significance in the univariate model were included in a multivariate model to derive adjusted odds ratios and build the final model. Data corresponding to odd and even years (2 subsets) were analyzed separately and used in a simple cross-validation.

**Results:**

Univariate analysis indicated that travel profile, prophylactic use, ethnicity, and province of birth were all associated with AMS in both subsets. In multivariate analysis, young men who traveled from lower altitude (600-800 m *vs*. 1300-1500 m, adjusted odds ratio (AOR) = 1.32-1.44) to higher altitudes (4100-4300 m *vs*. 2900-3100 m, AOR = 3.94-4.12; 3600-3700 m *vs*. 2900-3100 m, AOR = 2.71-2.74) by air or rapid land transport for emergency mission deployment (emergency land deployment *vs*. normal land deployment, AOR = 2.08-2.11; normal air deployment *vs*. normal land deployment, AOR = 2.00-2.20; emergency air deployment *vs*. normal land deployment, AOR = 2.40-3.34) during the cold season (cold *vs*. warm, AOR = 1.25-1.28) are at great risk for developing AMS. Non-Tibetan male soldiers (Tibetan *vs*. Han, AOR = 0.03-0.08), born and raised in lower provinces (eastern *vs*. northwestern, AOR = 1.32-1.39), and deployed without prophylaxis (prophylactic drug *vs*. none, AOR = 0.75-0.76), also represented a population at significantly increased risk for AMS. The predicted model was built; the area under receiver operating characteristic curve was 0.703.

**Conclusion:**

Before a group of young men first enter a high altitude area, it is important that a health service plan should be made referring to the group's travel profile and with respect to young men's ethnicity and province of birth. Low-cost Chinese traditional prophylactic drugs might have some effect on decreasing the risk of AMS, although this needs further verification.

## Background

Acute mountain sickness (AMS) is an important public health problem for highland newcomers [1]. Its symptoms, including headache, nausea, vomiting, and insomnia, typically appear 6-12 hours after arrival at high altitudes and dissipate within 4-7 days [2,3]. AMS is usually not life-threatening, but can seriously impact health quality, decrease productivity, and increase healthcare costs. In severe cases, AMS can lead to oliguria, retinal hemorrhage, ataxia, coma [4], and pulmonary and cerebral edema, which could threaten one's life. In 1960, hundreds of Chinese soldiers suffered malignant AMS during northern Tibet operations; the average mortality rate was 1-5%[5]. In the early 1990s, 10-20% of the air-ferried Chinese soldiers became completely incapacitated within the first days of arrival [6]. In recent years, a great number of young men have traveled to Tibet for business [7], mission and travel, including laborers, soldiers, and government officials. Most of them are at significant risk for developing AMS [8]. Thus, evaluating the AMS risk/protective factors have become a serious concern for the civil and military authorities in Tibet to aid in policy making for AMS treatment and prevention.

Many factors have been suggested as associated with AMS and may be useful for predicting risk of AMS. Some studies have assessed AMS risk by evaluating type and frequency of symptoms experienced by individuals during previous exposures [9,10], others have focused on early high respiratory rates and SaO_2 _values that are experienced during short-term exposure to hypoxic conditions [11,12]. However, these factors are dependent on pre-exposure to a hypoxic environment, which is not feasible for newly entering highland mass populations. Other physiological factors, such as heart rate variability [13], optic nerve sheath diameter, and intracranial pressure [14,15], were found to be associated with AMS. However, these factors occur at the individual level and are not directly helpful for macro-policy making or population level health intervention. Moreover, the data for some of these factors have not been consistent among different studies, such as heart rate variability [16], and their actual relevance remains controversial. Some biochemical and genetic factors have also been found to be associated with AMS [17-19]. However, detection of these is time consuming, costly, and requires advanced technical support, all features which limit their use in a mass population, especially in developing countries like China.

Successful AMS control should take population level factors into account. As some researchers have emphasized [20], an evaluation of the effect of population level factors on AMS can be used to generate more effective health-promotion and policies to control and prevent AMS from a macro-perspective. This will be helpful especially when large groups, such as soldiers or workers, are deployed under emergency situations. Therefore, assessing the risk factors at a population level, such as in the highland troops, would be applicable to cases of mass deployments to Tibet. However, very few studies to date have attempted this. Hence, this study aimed to evaluate some population level factors which are simple and accessible to estimate the odds ratio of AMS and build a prediction model based upon these factors. Although this method is not optimal in its precision, it will act as a form of reference for health service departments to guide reasonable health service for young men population entering high altitude regions.

## Methods

### Ethics statement

This study was approved by the Ethical Review Board of the Third Military Medical University. The requirement for informed consent was waived by the committee since we extracted data retrospectively from medical records. No intervention was performed between study researchers and individuals whose data were accessed and all data were anonymized prior to retrieval and analysis.

### Statistical analyses

**Figure 1:**
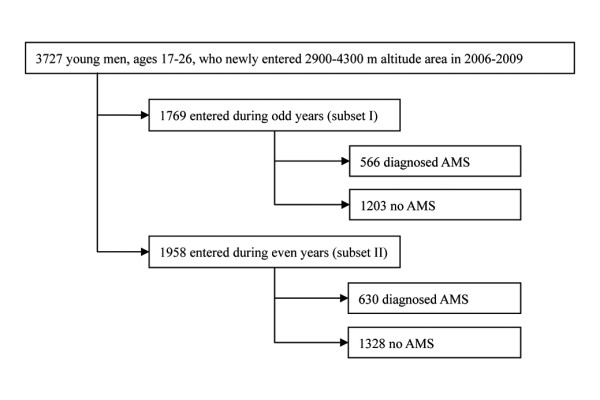
**Number of young men and partitioning of the data into two subsets according to entering year**.

For cross-validation the sample of data were partitioned into two complementary subsets (subset I, odd years; subset II, even years) according to their year of entry into the high altitude region and analyzed respectively as shown in Figure 1.

Logistic regression analysis was used to estimate the risk of AMS [21]; univariate logistic regression estimated the crude risk, and multivariate logistic regression estimated the adjusted risk. Univariate logistic regression analyses were also performed to calculate the odds ratios (ORs) of each factor on the risk for AMS. The significance and the lower and upper 95% confidence intervals (95%CI) were reported. The significance level in univariate analysis was two-tailed *p *< 0.10, and was a screening criterion to find candidate independent variables for entering into the multiple analysis. Multivariate logistic regression analyses were performed to calculate the adjusted odds ratios (AORs) and build the AMS probability model for the young men population. The fulfillment of independence and collinearity were tested in the multivariate models. The fit of the multiple logistic regression models was evaluated by the Hoshmer-Lameshow goodness-of-fit test. The discriminative power of significant predictive factors was tested using receiver operating characteristic (ROC) curves, including the area under the curve, with 95%CI. The regression analyses were performed by using SPSS 13.0 software (Chicago, IL, USA). The ROC curve analyses were performed using Med Calc 11.6 software (Belgium). In multivariate analysis, a two-tailed *p *< 0.05 was considered statistically significant.

### Data collection and inclusion criteria

Relevant data were obtained from the Health Service Department of the Chinese highland forces. Medical records of all young men who entered the Tibet-Qinghai Plateau during the years 2006-2009 were considered. The records had been originally collected by the respective Health Service Departments (the health management division of each troop) in Tibet. Because the data were from troops in different military operations, a few items in the health records differed, so we sometimes classified data according to the overall indications (for example, if data were presented as qualitative 'yes or no' in some records and qualitative measurements in others to indicate extent of severity of 'yes or no' status). The demographic characteristics, routine clinical parameters, and disease history were extracted from individual's medical record sheets. The travel profile was taken from the medical log file. Generally, personal information on the health record sheets was self-reported by the young men, while military medical officers entered the clinical information, and the leader of the health service group completed the medical log file. We excluded cases in which study data were missing.

An individual's data were included in this study if the young man: 1) had entered an area of 2900-4300 m and stayed there for at least two weeks; 2) had traveled to the high altitude location by vehicle or airplane; and 3) was born and had been raised in a region < 2500 m altitude. Individuals were excluded from this study if they had: 1) other acute diseases when they entered the high altitude area; 2) any severe chronic disease; 3) served in a highland region previously; 4) slept in a place higher than their destination during their trip; 5) passed through an area higher than their destination for more than four hours during their trip; or 6) incomplete study information. Any records that met the above criteria but represented an ethnicity group that composed less than 1% of the total medical records under consideration were also excluded. A total of 3727 young men's health records were included in this study. The range of ages was 17 to 26 years old (19.58 ± 1.34). The ethnic distribution was 93.16% Han (ethnic Chinese), 4.51% Hui (Muslim), and 2.33% Tibetan (who came from areas < 2500 m in the Sichuan or Gansu provinces). The diagnosis of AMS was based on the "Diagnostic Criteria of High Altitude Disease in China", which was brought into effect in China during the 1990s and introduced to the world through translation by John B. West in 2010 (Table 1) [22]. Unlike the Lake Louis self-report score, the clinical assessment of AMS was performed by the military medical officer. In many cases, the observers only recorded a qualitative conclusion of AMS, without a score. Thus, this study considered AMS as a binomial variable. If a young man was diagnosed with AMS of any severity during early highland exposure (within about 7 days), he was considered positive for AMS.

**Table 1:** Diagnostic criteria for acute mountain sickness in China.

Symptoms	Degree	Score
**Headache**		
1. No noticeable headache, no suffering expression, no effect on daily activity.	±	1
2. Mild headache with suffering expression, obvious improvement of headache after taking regular analgesic medicine, no effect on daily activity.	+	2
3. Moderate headache with suffering expression, slight improvement of headache after taking regular analgesic medicine, daily activity is affected.	++	4
4. Severe and unbearable headache, lies in bed and can not get up, no effect from regular analgesic medication.	+++	7
**Vomiting**		
1. Vomiting 1 to 2 times a day, vomit contains only intake food, obvious improvement with regular anti-emetic medication, no effect on daily activity.	+	2
2. Vomiting 3 to 4 times a day, final vomit contains gastric juice, slight improvement with anti-emetic medication, daily activity is affected.	++	4
3. Vomiting more than 5 times a day, must lie in bed and cannot get up, no improvement with regular anti-emetic medication.	+++	7
**Other**		
Dizziness/light-headedness, nausea, palpitation, shortness of breath, chest distress, dazzling/blurred vision, sleeplessness (insomnia), anorexia, abdominal distension, diarrhea, constipation, cyanosis of the lips, lethargy, and numbness of the extremities		1 point each

* a person with headache+, or vomiting+; or total score of 5 points or more was diagnosed AMS.

The following 13 factors, used as independent variables, were examined as risk factors for AMS: age, ethnicity, province of birth, education, body mass index (BMI), military rank, alcohol drinking, cigarette smoking, prophylaxis, start point altitude, destination altitude, deployment type, and travel season. These variables were chosen based on their status as recognized or hypothesized risk factors of AMS [23-25] and the fact that they were easily obtainable in an organized mass population. Age (scored by years) was handled as a continuous variable. Start point altitude, destination altitude, deployment type, travel season, prophylaxis, ethnicity, province of birth, education, military rank, alcohol drinking, and cigarette smoking were handled as categorical variables.

For each factor, two to four coded categories were established. Ethnicity was classified as Han, Tibetan, and Hui, as above mentioned. Because provinces in western China, except for Tibet and Qinghai, have an average altitude of 500-2500 m and those in the east are < 500 m, we divided the young men by province birthplace and residence (either east or west). We then subdivided the west province individuals into northwest and southwest groups according to the distinct climates of each region (temperate continental climate vs. subtropical monsoon climate, respectively). The northwest provinces included Xinjiang, Inner Mongolia, Shanxi, Gansu, and Ningxia; the southwest provinces included Sichuan, Yunnan, and Guizhou. Since the sample size for the east provinces was relatively small (n = 917) we did not subdivide by climate. Education was divided among ≤junior high school, high school, and ≥college. According to the World Health Organization's classifications, the BMI was divided into three groups: underweight (< 18.5), normal (18.5-24.9), and overweight (25.0-29.9)[26]. Military rank was classified as private, non-commissioned officer (NCO), and officer. Owing to the selection criteria of no previous high altitude (> 2500 m) exposure, the officers and NCOs included in this study were young and of low rank requiring no further subdivision. Both alcohol drinking and cigarette smoking were classified as "yes" (current drinking or smoking) or "no" (currently do not drink or currently do not smoke). The administration of a prophylactic was extracted from medical log file that recorded the data at the group level. It was scored as "yes" or "no". According to the records, in normal deployment, doses of rhodiola (0.6-1.2 mg/day) were administered one week prior to ascent, while in emergency deployment, rhodiola (0.6-1.2 mg/day) was administered as early as possible. During ascent, soldiers were administered danshen (0.3 mg/day). These drugs have been approved by the China State Food and Drug Administration and are routinely used throughout the country in both military and civilian populations for prevention and treatment of AMS. Both of the drugs are extracted from Chinese traditional herbs and provided in pill or capsule form with normal oral intake. Prophylaxis with other medicines, which were cited in very few cases, were excluded from this study. Start point altitude was divided among three groups: 600-800 m, 900-1100 m, and 1300-1500 m. Destination altitude was also classified among three groups: 2900-3100 m, 3500-3700 m, and 4000-4300 m. We chose to divide altitude into these groups since most of the data were concentrated in these intervals and easily distinguishable from one another. Deployment type included normal land deployment, emergency land deployment, normal air deployment, and emergency air deployment. For normal deployment, no missions were carried out within the first week of entering, as this time was used for rest and acclimation. For normal land deployment, all young men were given several days of rest after climbing for one day. For emergency land deployment, missions began immediately upon arrival after climbing, and there was no acclimation or rest period. For emergency air deployment, the situation was similar to emergency land deployment. Travel seasons were classified as either warm or cold. The warm season included June, July, and August, while the cold season included November, December, and March. There were not enough records of young men entering the plateau in the extremely cold months (January and February) to merit inclusion in the study. The characteristics of the young men used in this study are listed in Table 2.

**Table 2:** Characteristics of the young men exposed to high altitude in Tibet, 2006-2009

Indicators	Number	Percent(%)
**Demographic characteristics**		
**Male**	3727	100
**Age(y)**		
< 18	133	4
18-20	2813	75
21-26	781	21
**Ethnicity**		
Han (Chinese)	3472	93
Tibetan	87	2
Hui (Muslim)	168	5
**Birth province**		
Northwest province	1544	41
Southwest province	1266	34
East province	917	25
**Education**		
≤Junior high school	2060	55
= High school	1589	43
≥College	78	2
**BMI**		
Normal	2892	78
Underweight	426	11
Overweight	409	11
**Rank**		
Private	3173	85
NCO	433	12
Officer	121	3
**Health behavior**		
**Drinking alcohol**		
Yes	1155	31
No	2572	69
**Cigarette smoking**		
Yes	2242	60
No	1485	40
**Prophylaxis**		
No	2360	63
Yes	1367	37
**Travel profile**		
**Start point altitude**		
1300-1500 m	1449	39
900-1100 m	1074	29
600-800 m	1204	32
**Destination altitude**		
2900-3100 m	1245	33
3600-3800 m	1221	33
4100-4300 m	1261	34
**Deployment type**		
Normal by land	1706	46
Emergency by land	1142	31
Normal by air	500	13
Emergency by air	379	10
**Season**		
Warm	2133	57
Cold	1594	43

## Results

The descriptive analysis of the odd years data group, along with results of the univariate logistic regression, which was used to determine the variables status as potential risk or protective factors for the disease, are given in Table 3, and that for the even years group are in Table 4.

**Table 3:** Acute mountain sickness among young men who entered the Tibetan-Qinghai plateau: prevalence and univariate logistic regression analysis (odd years, n = 1769)

Indicators	Total number of young men	Number of diagnosed AMS cases(%)	β	OR	95%CI	*p*-value
**Demographic characteristic**						
**Age**	--	--	-0.01	0.99	0.92-1.07	0.781
**Ethnicity**						
Han (Chinese) (ref.)	1654	545(24.6)		1.00		
Tibetan	44	1(1.2)	-3.05	0.05	0.01-0.34	0.003
Hui (Muslim)	71	20(19.6)	-0.23	0.80	0.47-1.35	0.401
**Birth province**						
Northwest province (ref.)	726	228(22.9)		1.00		
Southwest province	595	176(21.0)	-0.09	0.92	0.72-1.16	0.474
East province	448	162(28.3)	0.21	1.24	0.97-1.59	0.093
**Education**						
≤Junior high school (ref.)	981	310(23.1)		1.00		
= High school	746	244(24.3)	0.05	1.05	0.86-1.29	0.625
≥College	42	12(20.0)	-0.14	0.87	0.44-1.71	0.679
**BMI**						
Normal	1375	449(32.7)				
Underweight	215	60(27.9)	-0.23	0.80	0.58-1.10	0.166
Overweight	179	57(31.8)	-0.04	0.96	0.69-1.35	0.828
**Rank**						
Private (ref.)	1507	487(23.9)		1.00		
NCO	204	59(20.3)	-0.16	0.85	0.62-1.18	0.329
Officer	58	20(26.3)	0.10	1.10	0.63-1.91	0.729
**Health behavior**						
**Alcohol drinking**						
Yes (ref.)	538	174(23.9)		1.00		
No	1231	392(23.4)	-0.02	0.98	0.79-1.21	0.836
**Cigarette smoking**						
Yes (ref.)	1052	354(25.4)		1.00		
No	717	212(21.0)	-0.19	0.83	0.67-1.02	0.071
**Prophylaxis**						
No (ref.)	998	341(26.0)		1.00		
Yes	771	225(20.6)	-0.23	0.79	0.65-0.97	0.026
**Travel profile**						
**Start point altitude**						
1300-1500 m (ref.)	606	166(18.9)		1.00		
900-1100 m	511	163(23.4)	0.22	1.24	0.96-1.61	0.100
600-800 m	652	237(28.6)	0.41	1.51	1.19-1.92	0.001
**Destination altitude**						
2900-3100 m (ref.)	558	91(19.5)		1.00		
3600-3700 m	587	202(26.2)	0.99	2.69	2.03-3.57	< 0.001
4100-4300 m	624	273(38.9)	1.38	3.99	3.03-5.25	< 0.001
**Deployment type**						
Normal by land (ref.)	797	118(15.4)		1.00		
Emergency by land	566	216(30.9)	0.69	2.00	1.58-2.53	< 0.001
Normal by air	240	90(30.0)	0.66	1.94	1.43-2.65	< 0.001
Emergency by air	166	72(38.3)	0.91	2.48	1.75-3.51	< 0.001
**Travel season**						
Warm (ref.)	998	301(21.6)		1.00		
Cold	771	265(26.2)	0.19	1.21	0.99-1.48	0.060

**Table 4:** Acute mountain sickness among young men who entered the Tibetan-Qinghai plateau: prevalence and univariate logistic regression analysis (even years, n = 1958)

Indicators	Total number of young men	Number of diagnosed AMS cases(%)	β	OR	95%CI	*p*-value
**Demographic characteristics**						
**Age**	--	--	0.05	1.05	0.98-1.13	0.148
**Ethnicity**						
Han (Chinese) (ref.)	1818	596(24.4)		1.00		
Tibetan	43	2(2.4)	-2.30	0.10	0.02-0.41	0.002
Hui (Muslim)	97	32(24.6)	0.01	1.01	0.65-1.56	0.966
**Birth province**						
Northwest province (ref.)	818	224(21.3)		1.00		
Southwest province	671	214(23.4)	0.10	1.10	0.88-1.37	0.391
East province	469	172(29.0)	0.31	1.36	1.07-1.73	0.012
**Education**						
≤Junior high school (ref.)	1079	351(24.1)		1.00		
= High school	843	270(23.6)	-0.02	0.98	0.81-1.19	0.815
≥College	36	9(16.7)	-0.37	0.69	0.32-1.49	0.344
**BMI**						
Normal	1500	480(32.0)				
Underweight	234	65(27.8)	-0.20	0.82	0.60-1.11	0.196
Overweight	224	85(37.9)	0.26	1.30	0.97-1.74	0.078
**Rank**						
Private (ref.)	1666	533(23.5)		1.00		
NCO	229	70(22.0)	-0.07	0.94	0.69-1.26	0.664
Officer	63	27(37.5)	0.47	1.59	0.96-2.65	0.073
**Health behavior**						
**Alcohol drinking**						
Yes (ref.)	617	194(22.9)		1.00		
No	1341	436(24.1)	0.05	1.05	0.86-1.29	0.638
**Cigarette smoking**						
Yes (ref.)	1190	392(24.6)		1.00		
No	768	238(22.5)	-0.09	0.91	0.75-1.11	0.367
**Prophylaxis**						
No (ref.)	1362	463(25.8)		1.00		
Yes	596	167(19.5)	-0.28	0.76	0.61-0.93	0.009
**Travel profile**						
**Start point altitude**						
1300-1500 m (ref.)	843	250(21.1)		1.00		
900-1100 m	563	188(25.1)	0.17	1.19	0.95-1.50	0.138
600-800 m	552	192(26.7)	0.24	1.27	1.01-1.59	0.044
**Destination altitude**						
2900-3100 m (ref.)	687	119(10.5)		1.00		
3600-3700 m	634	232(28.9)	1.01	2.75	2.13-3.56	< 0.001
4100-4300 m	637	279(39.0)	1.31	3.72	2.89-4.79	< 0.001
**Deployment type**						
Normal by land (ref.)	885	203(14.9)		1.00		
Emergency by land	587	221(30.2)	0.71	2.03	1.61-2.55	< 0.001
Normal by air	268	105(32.2)	0.77	2.16	1.62-2.90	< 0.001
Emergency by air	218	218(43.2)	1.06	2.90	2.13-3.95	< 0.001
**Travel season**						
Warm (ref.)	1135	346(21.9)		1.00		
Cold	823	284(26.3)	0.18	1.20	0.99-1.45	0.060

For the demographic factors, only ethnicity (OR = 0.05, Tibetan *vs*. Han, *p *< 0.10) and province of birth (OR = 1.24, east province *vs*. northwest province, *p *< 0.10) were significantly associated with AMS in the odd years data set. In the even years data set, however, province of birth (OR = 1.36, east province *vs*. northwest province, *p *< 0.10), ethnicity (OR = 0.10, Tibetan *vs*. Han, *p *< 0.10), BMI (OR = 1.30, overweight *vs*. normal, *p *< 0.10), and military rank (OR = 1.59, officer *vs*. private, *p *< 0.10) were significantly associated with AMS.

Among the health behavior factors, a protective effect was found from the administration of prophylaxis (OR_odd _= 0.79 and OR_even _= 0.76, yes *vs*. no, *p *< 0.10). No cigarette smoking was also identified as protective, but only in the odd years data set (OR_odd _= 0.83, yes *vs*. no, *p *< 0.10). The consumption of alcohol was not a significant contributor.

In the odd years data set, all of the travel factors examined were found to be significantly associated with AMS. Young men were significantly more likely to develop AMS if they: 1) went to a higher altitude (OR = 2.69, 3600-3700 m *vs*. 2900-3100 m, *p *< 0.10; OR = 3.99, 4100-4300 m *vs*. 2900-3100 m, *p *< 0.10); 2) came from a lower start point altitude (OR = 1.51, 600-800 m *vs*. 1300-1500 m, *p *< 0.10); 3) went to the highland in an emergency situation or by air (OR = 2.00, emergency by land *vs*. normal by land, *p *< 0.10; OR = 1.94 normal by air *vs*. normal by land, *p *< 0.10; OR = 2.48, emergency by air *vs*. normal by land, *p *< 0.10); or 4) traveled in a cold season (OR = 1.21, cold season *vs*. warm, *p *< 0.10). The analysis of even years data yielded similar results: (OR = 2.75, 3600-3700 m *vs*. 2900-3100 m, *p *< 0.10; OR = 3.72, 4100-4300 m *vs*. 2900-3100 m, *p *< 0.10; OR = 1.27, 600-800 m *vs*. 1300-1500 m, *p *< 0.10), (OR = 1.20, cold season *vs*. warm, *p *< 0.10), (OR = 2.03, emergency by land *vs*. normal by land, *p *< 0.10; OR = 2.16 normal by air *vs*. normal by land, *p *< 0.10; OR = 2.90, emergency by air *vs*. normal by land, *p *< 0.10), respectively.

Those independent variables having univariate significance (*p *< 0.10) were included in the multivariate logistic regression model. Then BMI, cigarette smoking, and military rank lost significance (*p *> 0.05); thus, these three variables were excluded from subsequent multiple regression models. When the multiple regressions were re-run, start point altitude, destination altitude, deployment type, travel season, prophylaxis, ethnicity, and province of birth retained significance in both data subsets (Table 5).

**Table 5:** Multivariate logistic regression to determine relationship between risk factors and AMS prevalence

Indicators		Odd years		(n = 1769)		Even years		(n = 1958)
	
	β	AOR	95%CI	*p*-value	β	AOR	95%CI	*p*-value
**Demographic indicators**								
**Ethnicity**								
Han (Chinese) (ref.)		1.00				1.00		
Tibetan	-3.39	0.03	0.01-0.25	0.001	-2.55	0.08	0.02-0.33	0.001
Hui	-0.29	0.75	0.43-1.31	0.309	0.06	1.06	0.66-1.69	0.810
**Birth province**								
Northwest province (ref.)		1.00				1.00		
Southwest province	-0.04	0.96	0.75-1.24	0.758	0.18	1.19	0.94-1.51	0.149
East province	0.33	1.39	1.07-1.81	0.015	0.28	1.32	1.02-1.70	0.034
**Health behavior**								
**Prophylaxis**								
No (ref.)		1.00				1.00		
Yes	-0.29	0.75	0.60-0.93	0.008	-0.28	0.76	0.60-0.94	0.014
**Travel indicators**								
**Start point altitude**								
1300-1500 m (ref.)		1.00				1.00		
900-1100 m	0.21	1.23	0.94-1.62	0.136	0.19	1.20	0.94-1.54	0.135
600-800 m	0.36	1.44	1.11-1.85	0.005	0.28	1.32	1.03-1.68	0.026
**Destination altitude**								
2900-3100 m (ref.)		1.00				1.00		
3600-3700 m	1.00	2.71	2.03-3.62	< 0.001	1.01	2.74	2.11-3.56	< 0.001
4100-4300 m	1.42	4.12	3.10-5.46	< 0.001	1.37	3.94	3.04-5.11	< 0.001
**Deployment type**								
Normal by land (ref.)		1.00				1.00		
Emergency by land	0.75	2.11	1.65-2.70	< 0.001	0.73	2.08	1.64-2.64	< 0.001
Normal by air	0.69	2.00	1.44-2.76	< 0.001	0.79	2.20	1.62-2.98	< 0.001
Emergency by air	0.88	2.40	1.66-3.46	< 0.001	1.21	3.34	2.41-4.65	< 0.001
**Travel season**								
Warm (ref.)		1.00				1.00		
Cold	0.25	1.28	1.04-1.59	0.022	0.22	1.25	1.02-1.53	0.034

In the multivariate models, Tibetan ethnicity was associated with significantly lower AMS risk than Han ethnicity (AOR_odd _= 0.03, 95%CI 0.01-0.25; AOR_even _= 0.08, 95%CI 0.02-0.33, *p *< 0.05); there was no difference in risk found between Hui (Muslim) and Han (ethnic Chinese). Birth in eastern provinces was associated with significantly higher AMS incidence than birth in northwestern provinces (AOR_odd _= 1.39, 95%CI 1.07-1.81; AOR_even _= 1.32, 95%CI 1.02-1.70, *p *< 0.05); there was no significant difference in risk found between birth in southwestern and northwestern provinces. Prophylaxis use was correlated to lower AMS incidence than non-use (AOR_odd _= 0.75, 95%CI 0.60-0.93; AOR_even _= 0.76, 95%CI 0.60-0.94, *p *< 0.05). The start point of 600-800 m was determined to be a significantly stronger AMS risk factor than the 1300-1500 m start point (AOR_odd _= 1.44, 95%CI 1.11-1.85; AOR_even _= 1.32, 95%CI 1.03-1.68, *p *< 0.05); there was no significant AMS risk difference between the start point of 900-1100 m and 1300-1500 m. The destination altitude of 4100-4300 m also represented a significantly stronger AMS risk factor and had more effect than the 2900-3100 m destination (AOR_odd _= 4.12, 95%CI 3.10-5.46; AOR_even _= 3.94, 95%CI 3.04-5.11, *p *< 0.05); meanwhile, the destination altitude of 3600-3700 m was associated with significantly higher risk than that of 2900-3100 m (AOR_odd _= 2.71, 95%CI 2.03-3.62; AOR_even _= 2.74, 95%CI 2.11-3.56, *p *< 0.05). Emergency land deployment was associated with higher AMS incidence than was normal land deployment (AOR_odd _= 2.11, 95%CI 1.65-2.70; AOR_even _= 2.08, 95%CI 1.64-2.64, *p *< 0.05). Normal air deployment was associated with higher AMS incidence than was normal land deployment (AOR_odd _= 2.00, 95%CI 1.44-2.76; AOR_even _= 2.20, 95%CI 1.62-2.98, *p *< 0.05). Emergency air deployment was associated with higher AMS incidence than was normal land deployment (AOR_odd _= 2.40, 95%CI 1.66-3.46; AOR_even _= 3.34, 95%CI 2.41-4.65, *p *< 0.05). Finally, travel in the cold season contributed stronger AMS risk than travel in the warm season (AOR_odd _= 1.28, 95%CI 1.04-1.59; AOR_even _= 1.25, 95%CI 1.02-1.53, *p *< 0.05). Then, the regression model equations of odd years dataset (Model I) and even years dataset (Model II) were derived, wherein the expected probability of each model in its training data were recorded for ROC analysis:

Model I:

p=1/[1+exp(−3.39X1+0.33X2−0.29X3+0.36X4+1.00X5+1.42X6+0.75X7+0.69X8+0.88X9+0.25X10)]

Model II:

p=1/[1+exp(−2.55X1+0.28X2−0.28X3+0.28X4+1.01X5+1.37X6+0.73X7+0.79X8+1.21X9+0.22X10)]

n both models, X_1_: Tibetan (value 0 or 1), X_2_: birth province (east), X_3_: prophylactic use, X_4_: start point (600-800 m), X_5_: destination (3600-3700 m), X_6_: destination (4100-4300 m), X_7_: transportation (emergency by land), X_8_: transportation (normal by air), X_9_: transportation (emergency by air), X_10_: season. Variables' range value: 0 or 1.

The Hosmer-Lemeshow tests revealed that both models fit well (Model I: *p *= 0.331, Model II: *p *= 0.068). The VIF of independent variables indicated an acceptable absence of collinearity (VIF < 0.50). Then, a 2-fold cross-validation was performed. Model I cross-validation was performed on dataset II, followed by Model II cross-validation on dataset I. The result of expected probability of each model was recorded for ROC analysis. The ROC curves for each model's training data and testing data were drawn. In Model I's training data, the area under the ROC curve was 0.705 (*p *< 0.001, 95%CI 0.683-0.726), while in its testing data, the area under ROC curve was 0.701 (*p *< 0.001, 95%CI 0.679-0.723); there was no significant difference between the two curves (*z *= 0.061, *p *= 0.951). In Model II's training data, the area under the curve was 0.709 (*p *< 0.001, 95%CI 0.685-0.733), while in its testing data, the area under ROC curve was 0.707 (*p *< 0.001, 95%CI 0.683-0.731); again, the two curves were not significantly different (*z *= 1.209, *p *= 0.227). These findings indicated that both models were significant and fit well; however, Model II seemed more appropriate as it had a higher area under curve in cross-validation than Model I.

## Discussion

This study found a correlation between AMS and the following risk factors at the population level: travel profile (start point altitude, destination altitude, deployment type, and travel season), demographic characteristics (ethnicity, born province), and prophylaxis.

The most important risk factors for AMS are rate of ascent and altitude reached [3]. There are two variables that are known to determine ascent rate; one is the ascent altitude and the other is ascent time. In the study presented herein, both of these variables were related to the particular travel characteristics we examined. Start point altitude and destination altitude were used to determine the value of ascent, while transportation method determined ascent time. A higher ascent rate occurs when someone comes from a lower start point and goes to a higher destination, or completes such travel in a shorter time. In this study, young men coming from the lowest start points had higher AMS rates than their counterparts from other (higher) start points. There was no significant difference identified between the second highest start point and the highest start point; this is in agreement with the notion that a higher start point (one closer to the destination point) may act as pre-exposure or sufficiently gradual ascent, both of which are considered effective methods to prevent AMS [3,10,27].

Type of deployment can also significantly affect the ascent rate, which in turn affects the risk of AMS. In our study, the risk of AMS was significantly higher for individuals experiencing air or emergency deployment, especially for those in emergency air deployment conditions. Gradual land transportation used in cases of normal deployment is represented by a slower ascent rate, while rapid land transportation, such as used in cases of emergency deployment, is represented by a faster ascent rate; air transportation always is represented by the fastest ascent rate. However, deployment type also is influenced by task status. Normal deployment is typical of a routine action, whereas emergency deployment is always indicative of an emergency operation, wherein soldiers deal with some task immediately upon arrival. The intensive labor itself causes hypoxia, which can cause greater severity and incidence of AMS in the early hours of exposure to high altitude [23].

High altitude stress is primarily due to hypoxia, which is a result of experiencing low atmospheric pressure. However, dry air and cold are also significant contributors to AMS development [28]. On the Tibet plateau, military transport is often conducted by truck, and the road conditions are less than optimal. The road quality is low-level when originally constructed and inadequate maintenance compounds the problems over time, causing even lower quality and further hindering timely pavement damage repair. Even the feeder (stem) roads are merely graveled in many regions, while the branch roads connecting frontier and remote units also generally gravel roads. These conditions cause serious bumps and jolting during land transport, which in turn can cause higher physiologic stress on the travelers using them. When young male Chinese soldiers travel to high altitudes by land during cold season months they are often exposed to bad weather, especially cold temperatures, high winds, and snow; cold season is also characterized by poor food support, which can sometimes be severely insufficient. The cold and dry weather further contribute to increased pulmonary-artery pressure and increase the risk of AMS [29]. Cold weather also increases the likelihood of developing respiratory infections [30], which can further contribute to the AMS condition.

In our study, young men who were born and raised in the east provinces of mainland China were more like to develop AMS. The main reason was determined to be the difference in altitude between the east (sea level) and west provinces (closer to the Tibet's average altitude). The results from our study indicate that individuals born in regions in the higher altitude level have an inherent advantage over those born in lower level, particularly in regards to less risk of developing AMS when traveling to Tibet. One possible reason for this is that young men who were born and raised in west provinces lived for long periods at relatively higher altitudes. This may have primed the individuals to the lower oxygen content of higher altitudes, which manifested as a factor for reduced risk of AMS. The two subdivided western groups (northwest and southwest) had no significant difference in AMS rate, suggesting that other regional factors, such as climate or social environment of the birth province, have no relationship with AMS. Further studies are needed to verify this presumption.

Ethnicity differences were found to significantly affect the development of AMS. It is well established that Tibetans are better adapted to high altitude and have a greater physical capacity in that environment than newcomers [7]. In our study, the individuals of Tibetan descent who were born and raised in a relatively low altitude area of China exhibited an obvious adaptive advantage over their Han and Hui counterparts. The Han and Hui groups had no significant difference between one another in terms of developing AMS. Therefore, our results are in line with the recent findings that Tibetans have genetically adapted to survive and thrive in a low oxygen atmosphere [31].

Many commercially available prophylactic drugs efficiently prevent AMS; these include the long-established acetazolamide and dexamethasone [32]. In China, rhodiola and danshen (extracted traditional Chinese medicine in pill or capsule from) are low cost and easily accessible; since they are also associated with desirable effectiveness and fewer contraindicates effects [33,34], they are frequently used to protect against hypoxia in mountain sickness in China's army. However, the prophylactic standards have yet to be established. Our study suggests that administration of prophylactics might decrease the incidence of AMS, although the effect was relatively small. Further randomized controlled trials to assess the efficacy of these prophylactic drugs are required to draw a firm conclusion on this matter.

Still other factors, such as BMI, smoking, and military rank were slightly associated with AMS by univariate analysis; however, in multivariate analysis the significance was lost. Other researchers have reported that obesity is a risk factor of AMS [35,36]; however, in our study, BMI did not significantly contribute to AMS. A possible explanation for this is that, unlike the previous studies, most of the young men used in our study were physically fit, probably due to the selection of recruit criteria for join the army and routine military training. Although a few of the individuals were slightly overweight, none were exceedingly overweight or fit the clinical definition of obese according to the World Health Organization's definition [26]. In addition, several previous studies have indicated that age is a contributing factor to AMS, children that have been exposed acutely to high altitude are more sensitive to hypobaric hypoxia than adults [37-39]. It was also reported that people > 50 years old show slightly decreased susceptibility to AMS than younger people [30,40,41]. However, in this study, age was not found to be significantly associated with AMS. One possible reason could be that all the persons in this study were young adults, and the age gap among them were not more than 10 years (75% of them had only 2 years age gap); the effect of age was, thus, not easy detected by current methods.

The results derived from this study can be used as a reference when planning an operation of a group of young men entering highland regions. Obviously, slowing down or limiting the ascent profile of people starting from lower regions, allowing time for acclimatization, and limiting the final altitude will be helpful for reducing AMS risk, if the operations allow. Young men whose homelands are in low altitude should be given more medical attention to detect early symptoms and given more time to acclimatize. In addition, administration of prophylaxis using Chinese traditional drugs prior to the initiation of the operation might be beneficial, although this needs further verification. If the operation or mission condition do not allow for decreased ascent rate, the AORs could be a quantitative reference when calculating the additional medical resources that should be prepared for the corresponding condition. For example, assuming all other conditions are equal, travel in the cold season should deploy 1/4 more medical resources than in warm season. The model established could be used as a tool that considers multiple factors to estimate AMS rate before a group of young men newly enters highland areas. The particular factors can be entered into the model to forecast the AMS rate before a group enters the highlands. This information could then assistant the authorities in preparing adequate medical resources, such as medicine, oxygen equipment, and accompanying physicians, to support the operation. It also could help the authorities to evaluate the work ability in the early time of the young men's group entering the highland and allow for arranging reasonable operations that may be achieved under the likely conditions. It is important to note the inherent limitations of a retrospective study (detailed below) restrict the design an absolutely accurate equation. Nevertheless, the newly developed statistical models could provide some timely information pertaining to the need for medical preparations aimed at a young men population newly entering highland areas. It also provides some valuable information applicable to designing future prospective research studies and health intervention strategies.

### Strengths and limitations

Our study was, to our knowledge, the first analysis of the relationship between multiple population level factors and AMS in China's highland troops, and also the first study aimed to assess risk of AMS for young adult Chinese males who newly entered high altitude regions (> 3000 m) by using simple and accessible population level factors which do not require complex or costly biochemical or physiological tests. This study had sufficient sample size to assess the impact of high altitude on AMS adjusting for the factors related to travel, demographics, and health behaviors which may affect AMS. The validity of AMS data from the military medical records is likely to be high, as the data were collected by trained investigators using rigorous criteria and the individual's health records were available (in most cases) for adjudication.

Several limitations existed in this study design and should be considered when generalizing our findings to other populations or conditions. First, in this study the AMS diagnosis criteria were based on the published "Diagnostic Criteria of High Altitude Disease in China". Thus, the incidence of AMS may be different according to the use of different scoring systems, such as the Lake Louise AMS Scoring System. Second, the study was carried out in a homogenous population group; all were young adult males, and most were in similar physical fitness (including BMI). Third, in this study, occupation, diet, and physical load at high altitude were negligible because everyone experienced the same conditions. Therefore, the identifiable risk factors for AMS among such a specialized population are limited. Moreover, the results were based on previously collected data that did not allow direct testing of causal effects. Precise AMS severity was not described in all the medical records, limiting the study's ability to deal with the outcome of AMS as all-or-none. Finally, AMS cases were diagnosed by different observers over a period of almost four years, so some bias is inevitable and uncontrollable; however, we presumed the sample size was large enough to, at least partially, limit this confounding effect.

## Conclusions

From a public health perspective, medical departments must deploy reasonable medical human resources, medicines, and equipment to efficiently cope with AMS. All these preparations are based on quantitative pre-evaluation of a certain population's risk of developing AMS. In this study, we estimated the AORs of AMS risk and built an AMS prediction model for a young men population newly entering highland areas. When planning a highland operation, the high risk factors of low altitude homeland, high ascent rate, and high ascent altitude, should be minimized or the traveler's give more time for acclimatization (if the deployment conditions allow such). Effective health service plans could be made according to the group's travel profile and their ethnicity and birth province structure (quantitatively estimated by the AORs and the newly developed model). The Chinese traditional hypoxia prophylaxis, rhodiola and danshen, might be beneficial, although this needs further verification. At this time, it is difficult to make an accurate equation to precisely predict AMS, due to some shortcomings of the study design; thus, further investigations including other populations will be necessary. Nevertheless, the findings from this study provide a valuable reference to guide highland health services and provide information for further study.

## Abbreviations

AMS: acute mountain sickness; 95%CI: 95% confidential interval; OR: odds ratio; AOR: adjusted odds ratio; NCO: non-commissioned officer; BMI: body mass index; receiver operating characteristic curves: ROC curves.

## Conflicting interests

The authors declare that they have no competing interests.

## Authors' contributions

YG and XL designed the study. XL collected and analyzed the data and drafted the manuscript. HY provided expert statistical advice. FT and YL collected the data. TP provided technical support. All authors were involved in critical evaluation and editing of the manuscript, and read and approved the final version.

## Pre-publication history

The pre-publication history for this paper can be accessed here:

http://www.biomedcentral.com/1471-2458/11/740/prepub
